# Stereotactic body radiotherapy using the forward-planned static-port tomotherapy for lung cancer: a novel planning technique with the newly-developed mode

**DOI:** 10.1093/jrr/rraa092

**Published:** 2020-09-18

**Authors:** Yoshihiko Manabe, Akifumi Miyakawa, Takuhito Kondo, Yuki Yamada, Seiji Hashimoto, Satoshi Ishikura, Yuta Shibamoto

**Affiliations:** Department of Radiology, Nagoya City University Graduate School of Medical Sciences, 1 Kawasumi, Mizuho-cho, Mizuho-ku, Nagoya 467-8601, Japan; Department of Radiation Oncology, Nanbu Tokushukai Hospital, 171-1 Hokama, Yaese-cho, Simajiri-gun, Okinawa 901-0493, Japan; Department of Radiology, Nagoya City University Graduate School of Medical Sciences, 1 Kawasumi, Mizuho-cho, Mizuho-ku, Nagoya 467-8601, Japan; Department of Radiology, Nagoya City University Graduate School of Medical Sciences, 1 Kawasumi, Mizuho-cho, Mizuho-ku, Nagoya 467-8601, Japan; Department of Radiology, Nagoya City University Graduate School of Medical Sciences, 1 Kawasumi, Mizuho-cho, Mizuho-ku, Nagoya 467-8601, Japan; Department of Radiation Oncology, Nanbu Tokushukai Hospital, 171-1 Hokama, Yaese-cho, Simajiri-gun, Okinawa 901-0493, Japan; Department of Radiology, Nagoya City University Graduate School of Medical Sciences, 1 Kawasumi, Mizuho-cho, Mizuho-ku, Nagoya 467-8601, Japan; Department of Radiology, Nagoya City University Graduate School of Medical Sciences, 1 Kawasumi, Mizuho-cho, Mizuho-ku, Nagoya 467-8601, Japan

**Keywords:** tomotherapy, stereotactic body radiotherapy, lung cancer, static-port tomotherapy, forward planning mode

## Abstract

With the newly-developed static-port forward-planning (FP) mode of tomotherapy, the ratio of the dose of the planning target volume (PTV) periphery to the maximum dose can be easily adjusted by modifying leaf margins when planning stereotactic body radiotherapy (SBRT). The purpose of this study was to evaluate the characteristics of FP plans compared to helical intensity-modulated radiotherapy (IMRT) and helical 3D conformal radiotherapy (3DCRT) plans of SBRT for lung tumors. The three plans were created for 14 tumors in 11 patients. For 13 tumors, 60 Gy in 7.5-Gy fractions was prescribed for a minimum coverage dose of 95% of the PTV (D95). The prescribed isodose line (PIL) was intended to be 60–80% of the maximum dose. Nine angles were used for the FP plans. The median D98 and D50 of the internal target volume for FP, helical-IMRT and helical-3DCRT plans were 70.4, 71.4 and 60.5 Gy, respectively (*P* < 0.001), and 77.7, 75.7 and 62.3 Gy, respectively (*P* < 0.0001). The median PIL and the lung volume receiving ≥20 Gy (V20) were 73.4, 73.4 and 94.3%, respectively (*P* < 0.0001), and 4.7, 4.0 and 5.7%, respectively (*P* < 0.0001). These parameters were not significantly different between the FP and helical-IMRT plans. The median beam-on times were 238.6, 418.9 and 197.1 s, respectively (*P* < 0.0001). The FP plans reduced the beam-on time by 43% compared to the helical-IMRT plans. The dose distribution of the FP plans was comparable to that of the helical-IMRT plans. The helical-3DCRT plans could not adjust PIL to be 60–80%.

## INTRODUCTION

Stereotactic body radiotherapy (SBRT) is now a popular treatment strategy for stage I and oligometastatic lung cancer [1–12]. In the planning of SBRT, it is important to increase the dose to the center of a tumor while delivering sufficient doses to the tumor periphery. The guidelines of the European Society for Radiotherapy and Oncology and other papers recommend that the isodose line on the periphery of the planning target volume (PTV) (prescribed isodose line, PIL) should be adjusted to 60–80% of the maximum dose for peripherally located tumors [1–5]. Promising results using a similar setting of PIL (60–90%, avoiding hot spots in organs at risk) for centrally located tumors have also been reported in a phase I/II trial [6].

**Fig. 1.**
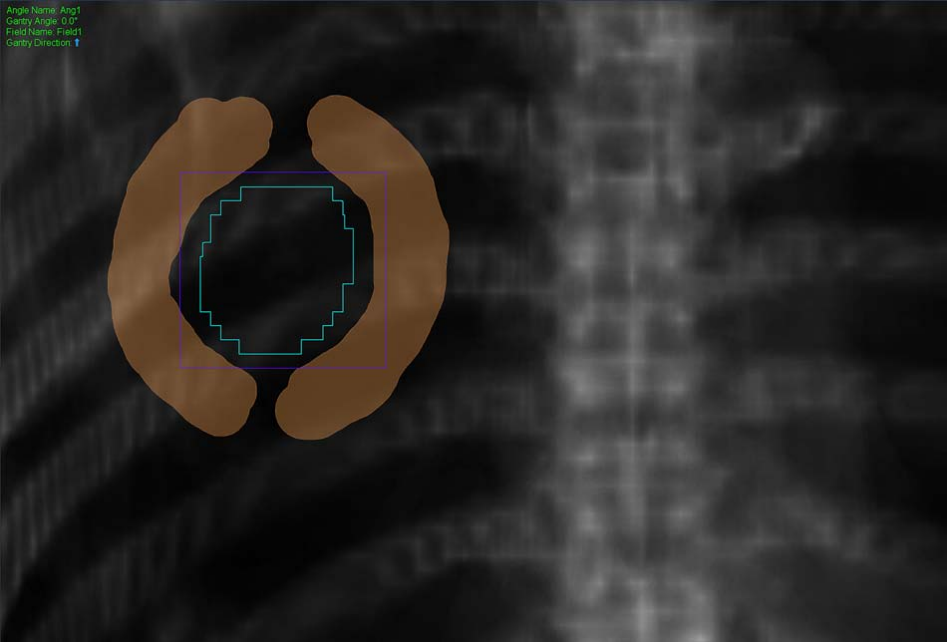
Brush tool (brown color) to modify leaf margin from each angle with digitally reconstructed radiography images in the FP mode.

TomoTherapy® (Accuray Inc., Sunnyvale, CA, USA) is a radiation delivery system for dynamic intensity-modulated radiation therapy (IMRT) that is also capable of delivering stereotactic radiotherapy [13–16]. Favorable outcomes of SBRT using tomotherapy have been reported for lung tumors [7, 8]. With conventional tomotherapy, a 3D conformal radiotherapy (3DCRT) mode is available. However, it is based on inverse planning, so the margins between the PTV and multileaf collimators (leaf margin) cannot be adjusted in 3DCRT mode. As a result, it has been difficult to adjust the ratio of the dose of the PTV periphery to the maximum dose using conventional tomotherapy with the 3DCRT mode.

**Table 1:** Patient and tumor characteristics

			*n*
Total patients			11
Total tumors			14^a^
Sex	Male/female		10/1
Age (years)	Median (range)		83 (64–87)
Tumor^b^	Stage I NSCLC/lung metastasis/local recurrence after surgery/local recurrence after chemotherapy for SCLC		8/4/1/1
Tumor site	Right/left upper lobe/middle (lingular) lobe/lower lobe peripheral zone/central zone		8/6
			7/2/5
			9/5
Volume	ITV	Median (range) (ml)	3.9 (0.5–25.3)
	PTV		16.2 (4.7–65.6)

^a^Three patients underwent stereotactic body radiotherapy twice.

^b^NSCLC = non-small cell lung cancer, SCLC = small cell lung cancer.

Recently, static-port forward-planning (FP) mode has been developed for clinical use, in addition to helical/static-port IMRT mode and helical/static-port 3DCRT mode. The static-port tomotherapy is named TomoDirect® and the FP mode is based on the TomoDirect®. Using the FP mode, leaf margins can be readily adjusted with the brush tool from each angle with digitally-reconstructed radiographic images (Fig. 1). For example, if we trace the surface of the 2 mm-expanded PTV with the brush tool, we can set the leaf margin to be 2 mm for the field. Actually, the corner of the leaf (width = 6.25 mm) is set along the surface of the 2 mm-expanded PTV, so the distance between the surface of the PTV and the leaf will be 2.0–6.25 mm in this situation. In our preliminary evaluation, the PIL could be adjusted with the FP mode during planning of SBRT for lung tumors. In this study, we evaluated the characteristics of FP plans compared to conventional helical-IMRT and helical-3DCRT plans of SBRT for lung tumors.

## MATERIALS AND METHODS

### Study approval and patients

This study was approved by our institutional review board (No. 2018–003). The study subjects were 11 patients aged 64–87 years (median, 83) with 14 lung tumors. Ten of them were men. All patients gave written informed consent before entry to the study. In all 14 tumors, three plans using the FP, helical-IMRT and helical-3DCRT modes were made and compared. Static-port tomotherapy is useful to reduce low-dose exposure to the lung when the target volume is large (e.g. locally advanced lung cancer), but the conformity deteriorates compared to helical tomotherapy [17]. In the situation of SBRT for lung tumors, the target volumes are small and the irradiated lung volumes tend to be small. Thus, we used helical-IMRT and helical-3DCRT plans for comparison. The patient characteristics are summarized in Table 1.

### Computed tomography simulation and planning

All patients were immobilized in a supine position with a vacuum bag system (BodyFIX; Medical Intelligence, Schwabmünchen, Germany) alongside the whole body. Our methods for immobilization were described in detail previously [9]. Axial non-contrast-enhanced computed tomography with a slice thickness of 2.5 mm was performed for treatment planning in the supine position under normal breathing and with breath-holding during the expiratory and inspiratory phases. Contouring of target volumes and normal structures was performed on the Pinnacle^3^ version 9 treatment planning system (Philips Medical System, Eindhoven, The Netherlands). The clinical target volume was defined as the visible gross tumor volume. The clinical target volumes on computed tomography during the three phases were superimposed on the Pinnacle^3^ to represent the internal target volume (ITV). We defined the PTV margin for the ITV as 5 mm in all directions. We defined the lung (excluding the ITV), rib, skin, spinal cord, esophagus, trachea, heart and great vessels as organs at risk. The contours created in the treatment planning system were exported to the tomotherapy treatment planning system (Precision version 1, Accuray, Inc., Sunnyvale, USA), where all plans were generated. This was a planning study, so the generated plans were not implemented in practice.

**Fig. 2.**
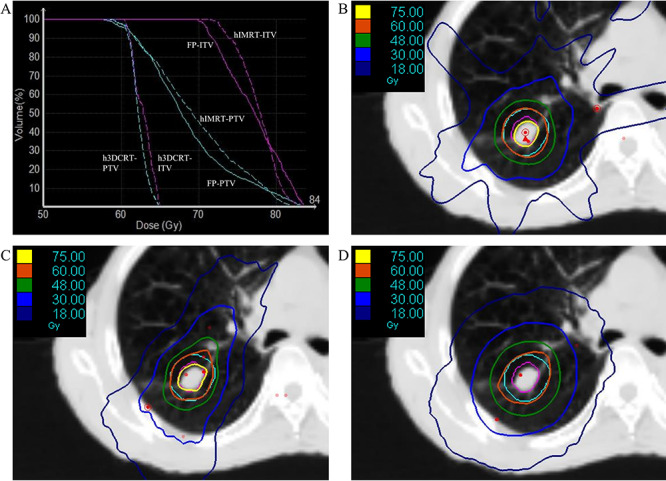
DVH curves for the ITV and PTV with the three modes (**A**). Dose distributions in the tumor using static-port forward planning (**B**), helical-IMRT (**C**) and helical-3DCRT mode (**D**). h = Helical.

For 13 of the 14 tumors, 5.0-cm dynamic jaws were used and 60 Gy in 7.5-Gy fractions was prescribed for a minimum coverage dose of 95% (D95) of the PTV. For the remaining tumor located near the heart, 60 Gy in 7.5-Gy fractions for D70 of the PTV was prescribed to fulfill the dose constraints in the FP and helical-IMRT plans. In this planning study, we included centrally located tumors only when the dose constraints were met for the FP and helical-IMRT plans (maximum to the spinal cord ≤32 Gy, maximum to the esophagus <40 Gy, maximum to the trachea ≤44 Gy, maximum to the heart ≤44 Gy, irradiated dose to 10 mL of the volume in the great vessels to 56 Gy). The same dose-fractionation was prescribed for peripherally located tumors (*n* = 9) and centrally located tumors (*n* = 5) to minimize dose variations. First, the FP plans were generated. In the FP plans, nine angles at even intervals (0, 40, 80, 120, 160, 200, 240, 280 and 320 degrees) were used. The PIL was intended to be 60–80% of the maximum dose. The leaf margin between the PTV and multi-leaf collimators was adjusted from 0 to 4 mm for each angle. When the helical-IMRT plans were generated, the maximum PTV doses and PILs were intended to be almost equal to those of the FP plans for each patient, but other dose–volume parameters of the FP plans were neglected to minimize the bias in the planning. First, the modulation factor was set to be 2.0, but it was difficult to increase the maximum dose of the PTV in some tumors. For those cases, we set the modulation factor to be 3.0. If all else failed, the modulation factor was set to be 5.0. In the helical-3DCRT plans, once the dose prescription (D95 of the PTV = 60 Gy) was set, the leaf margin was automatically set because it is based on inverse planning. Thus, the PIL could not be adjusted. A fine calculation grid (1.95 x 1.95 mm) was used for the final calculation process.

### Plan evaluation and statistical analysis

To compare the three plans, several indices were evaluated. Dose distribution in the targets and organs at risk and beam-on time were evaluated using the Friedman test for all three modes, and the Wilcoxon signed-rank test with Bonferroni correction was used to compare the FP and helical-IMRT modes. Statistical analyses were carried out with the software package ‘R’ [18]. All planning and evaluation was performed by one radiation oncologist (Y.M.).

## RESULTS

Figure 2 shows the dose-volume histogram (DVH) graphs and representative dose distributions for the three plans in a tumor. Figure 2D shows the dose distribution using the helical-3DCRT mode. The PTV (light blue line) is well covered by the 60-Gy line, but the maximum dose is only 65 Gy (Fig. 2A). Thus, the PIL is 92% (60/65 Gy). In contrast, the FP plan achieved greater dose concentration compared to the helical-3DCRT plan (Fig. 2B). The yellow line represents the 75-Gy line. The gross tumor volume is well covered by 75 Gy. The maximum dose is 84 Gy, so the PIL is 71% (60/84 Gy). The maximum dose can also be adjusted with the helical-IMRT mode (Fig. 2C), but the beam-on time was longer than for the other plans (FP: 264 s, helical-IMRT: 399 s, helical-3DCRT: 220 s).

The results regarding dose distribution and beam-on time of the three plans for the 14 tumors are summarized in Tables 2 and 3. The PILs of the helical-3DCRT plans were all >90% and could not be adjusted. The median D98 of the PTV was slightly lower in FP plans compared to helical-IMRT plans, but the median D50 of the PTV was superior in FP plans. The parameters of the ITV, median R50 (ratio of 50% prescription isodose volume to the PTV volume), D2cm (maximum dose at 2 cm from PTV in any direction) and the median percentage volume receiving 20 Gy (V20) of the lung were similar between the FP and helical-IMRT plans. The FP plans reduced the beam-on time by 43% compared to the helical-IMRT plans. Dose–volume parameters for organs at risk were not significantly different between the FP plans and helical-IMRT plans except for the maximum dose to the rib (Table 3).

**Table 2:** Treatment parameters, dose coverage for targets and beam-on times of the three plans

	FP	hIMRT^a^	h3DCRT^a^	*P* ^b^	*P* ^c^
	Median (range)				
Jaw size	5.0 cm (dynamic)			-	
Pitch	0.50 (0 .500–0.500)	0.215 (0.172–0.215)	0.430 (0.215–0.430)	-	-
Modulation factor	-	2.6 (2.0–5.0)	-	-	
Beam-on time (s)	238.6 (212.1–333.7)	418.9 (234.7–550.6)	197.1 (175.6–220.0)	<0.0001	0.026
ITV					
D98 (Gy)	70.4 (53.8–75.5)	71.4 (55.3–75.5)	60.5 (59.7–62.2)	<0.001	1.0
D50 (Gy)	77.7 (71.7–80.0)	75.7 (69.2–81.8)	62.3 (60.3–63.0)	<0.0001	0.27
D2 (Gy)	81.3 (75.0–84.7)	80.9 (74.4–84.5)	63.4 (61.7–64.8)	<0.0001	0.74
PTV					
D98 (Gy)	58.5 (38.8–60.0)	59.7 (40.5–60.6)	59.2 (57.9–60.0)	<0.001	<0.01
D50 (Gy)	71.2 (67.4–74.6)	68.9 (64.4–71.5)	62.1 (60.4–62.9)	<0.0001	<0.01
D2 (Gy)	80.5 (74.7–83.8)	79.6 (73.8–82.8)	63.3 (61.6–64.7)	<0.0001	0.092
PIL (%)	73.4 (70.4–79.9)	73.4 (70.2–79.8)	94.3 (92.3–96.3)	<0.0001	1.0
R50	5.8 (4.6–9.0)	5.3 (4.2–7.7)	8.2 (6.0–12.3)	<0.0001	1.0
D2cm (Gy)	32.6 (26.5–52.0)	31.7 (21.5–52.6)	36.6(27.2–46.4)	0.74	1.0

^a^hIMRT = helical-IMRT mode, h3DCRT = helical-3DCRT mode.

^b^ *P* Value among three plans*.*

^c^ *P* Value between FP and hIMRT plans.

**Table 3:** Dose–volume parameters of the organs at risk

	FP	hIMRT^a^	h3DCRT^a^	*P* ^b^	*P* ^c^
	Median (range)				
Lung V20Gy^a^ (%)	4.7 (2.1–9.6)	4.0 (1.9–8.6)	5.7 (2.7–10.7)	< 0.0001	0.53
Rib max (Gy)^d^	74.2 (22.3–82.0)	61.0 (21.6–78.5)	61.9 (27.6–65.6)	0.062	< 0.01
Skin max (Gy)^d^	19.0 (16.7–37.5)	16.5 (9.4–42.0)	20.1 (11.0–51.2)	0.26	0.72
Spinal cord max (Gy)^e^	9.1 (2.4–12.0)	8.1 (4.3–9.4)	10.3 (8.4–16.2)	0.041	1.0
Esophagus max (Gy)^e^	13.3(3.9–16.6)	8.6 (6.4–13.9)	15.3 (11.1–19.1)	0.022	1.0
Trachea max (Gy)^e^	16.7 (16.0–27.7)	14.1 (11.4–42.6)	25.5 (17.3–34.7)	0.091	1.0
Heart max (Gy)^e^	4.2 (0.4–43.6)	1.7 (0.3–44.0)	2.2 (0.4–61.0)	0.074	1.0
Great vessels					
D10ml Max (Gy)^a,e^	21.8 (7.0–37.9)	17.5 (12.6–32.6)	25.9 (22.0–45.9)	0.022	1.0

^a^hIMRT = helical-IMRT mode, h3DCRT = helical-3DCRT mode, V20Gy = percentage volume receiving 20 Gy, D10ml = irradiated dose to 10 mL of the volume.

^b^ *P* Value among three plans*.*

^c^ *P* Value between FP and hIMRT plans.

^d^For tumors located in peripheral zone (*n* = 9).

^e^For tumors located in central zone (*n* = 5).

## DISCUSSION

This study revealed that the FP plans achieved comparable dose distributions and reduced the beam-on time by 43% compared to the helical-IMRT plans for SBRT of lung tumors. Previous studies reported that a reduction of treatment time contributes to positioning accuracy for stereotactic intracranial and body radiotherapy [19, 20]. The beam-on times of IMRT plans were longer than those of the other plans. We considered that this was because IMRT is an aggregation of extra fine beams. When the dose of a fraction is high, the gantry periods can be >60 s, and thus the plan will not be able to be implemented in practice due to limitations of the system. To avoid this limitation of the gantry period, a smaller pitch and more rotations would be needed, extending the beam-on time. The helical-3DCRT plans achieved the shortest beam-on time, but could not adjust PIL to be 60–80%. It is reported that modification of leaf margins is useful to adjust PIL in the planning of SBRT for lung tumors using a conventional line-accelerator based machine [21]. Based on the results of present study, a similar technique using the FP mode was considered useful in tomotherapy for SBRT. To the best of our knowledge, evaluation of the FP mode for lung tumors has not yet been reported.

The median maximum dose in the rib was higher in the FP plans than in the helical-IMRT plans. The dose of the rib can be reduced by narrowing the leaf margin of the rib side for each angle. However, the hot spot will shift out of the center and the dose coverage for the ITV will deteriorate. When the tumor is located adjacent to the rib or other organs at risk (e.g. spinal cord, esophagus), this trade-off should be taken into consideration. In that situation, the helical-IMRT plan is a candidate if the long beam-on time is acceptable.

In clinical practice, it is an issue whether higher (near 80%) or lower (near 60%) PIL should be used. For example, when the prescribed peripheral dose is set to 60 Gy, the central maximum dose changes drastically from 75 Gy (PIL = 80%) to 100 Gy (PIL = 60%). Table 4 shows the clinical results between PIL = 60 and 80% in the same institution. Although different prescribed dose groups were included and direct comparison was not be available, the local control rate and overall survival rate seemed favorable in the series of PIL = 60%. However, grade ≥2 pulmonary toxicity rate was higher in the patients treated with PIL = 60%, although their planning study showed that the setting of PIL = 60% could decrease the lung dose while maintaining the target dose [21]. Further investigation would be warranted to determine the optimal PIL. When the PIL is low, there is concern about the interplay effect in the helical-IMRT plans because low-PIL plans have steep dose gradient. The FP mode does not use intensity modulation, which would be an advantage of the FP mode in the low-PIL SBRT.

**Table 4:** Clinical results of studies using different isodose prescription

Author/publication year	*n*	PIL	Operable	Tumor size	Dose	3 Year LC^a^	3 YearOS^a^	Grade ≥2 pulmonary toxicity
				<3/≥ 3 cm	(in 5 fractions)			
Takeda *et al.* [2] 2009	63	80%	14 (22%)	38/25	50 Gy	93%	73%	5%
Tsurugai *et al.* [3] 2019	250^b^	60%	66 (28%)	160/90	60 Gy (*n* = 66)	99%	78%	9.6%
					50 Gy (*n* = 157)			
					40 Gy (*n* = 27)			

^a^LC = Local control, OS = overall survival.

^b^250 Tumors in 237 patients.

Some points should be noted for SBRT planning using FP mode. First, the ITV method was used because no respiratory-gating or dynamic tumor-tracking system was available for tomotherapy at the time of this study. Thus, the PTV tended to be large when the tumor existed in the lung base. However, a real-time tumor-tracking system with tomotherapy has recently been developed [22]. Using this system, the volume of PTV can potentially be reduced. Second, the setup of the patients to implement SBRT plans with FP mode should be strictly controlled, because these plans have steep dose gradients. Slight errors in position can influence the clinical results.

In conclusion, the forward plans reduced the beam-on time by 43% compared to the helical-IMRT plans. The dose distribution of the forward plans was comparable to that of the helical-IMRT plans. The helical-3DCRT plans achieved the shortest beam-on time, but could not adjust the PIL to be 60–80%.
